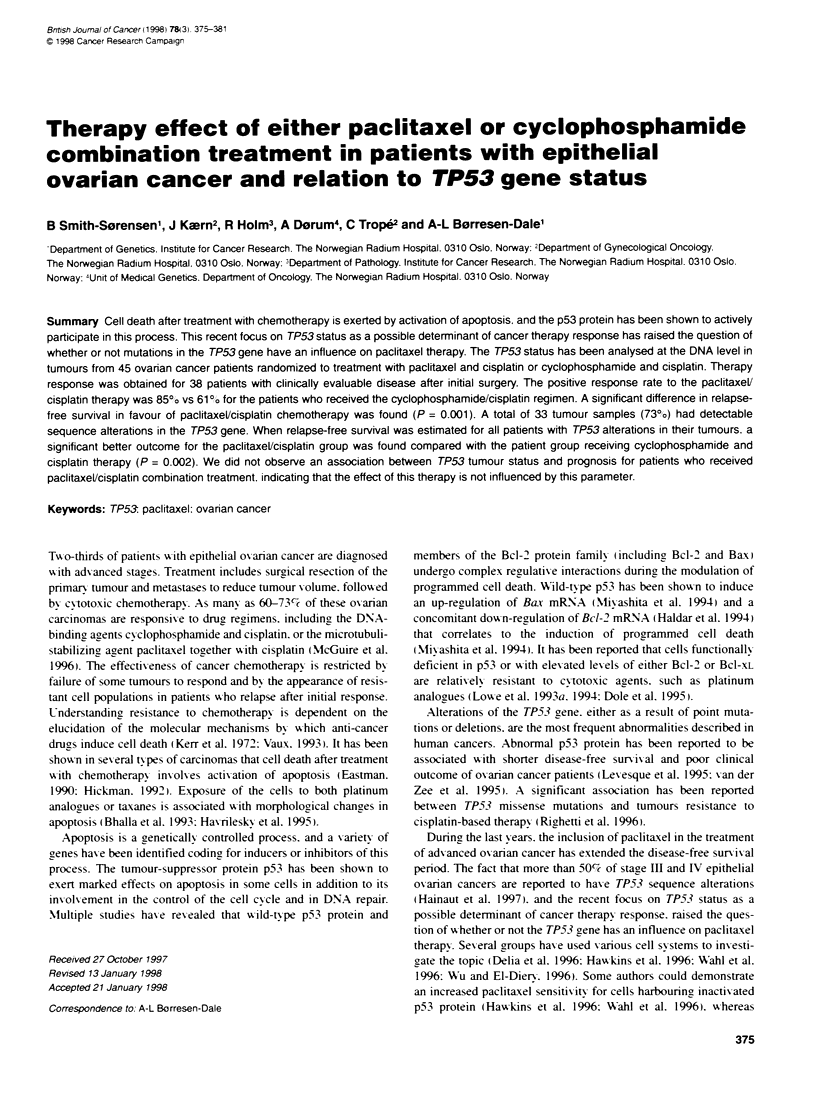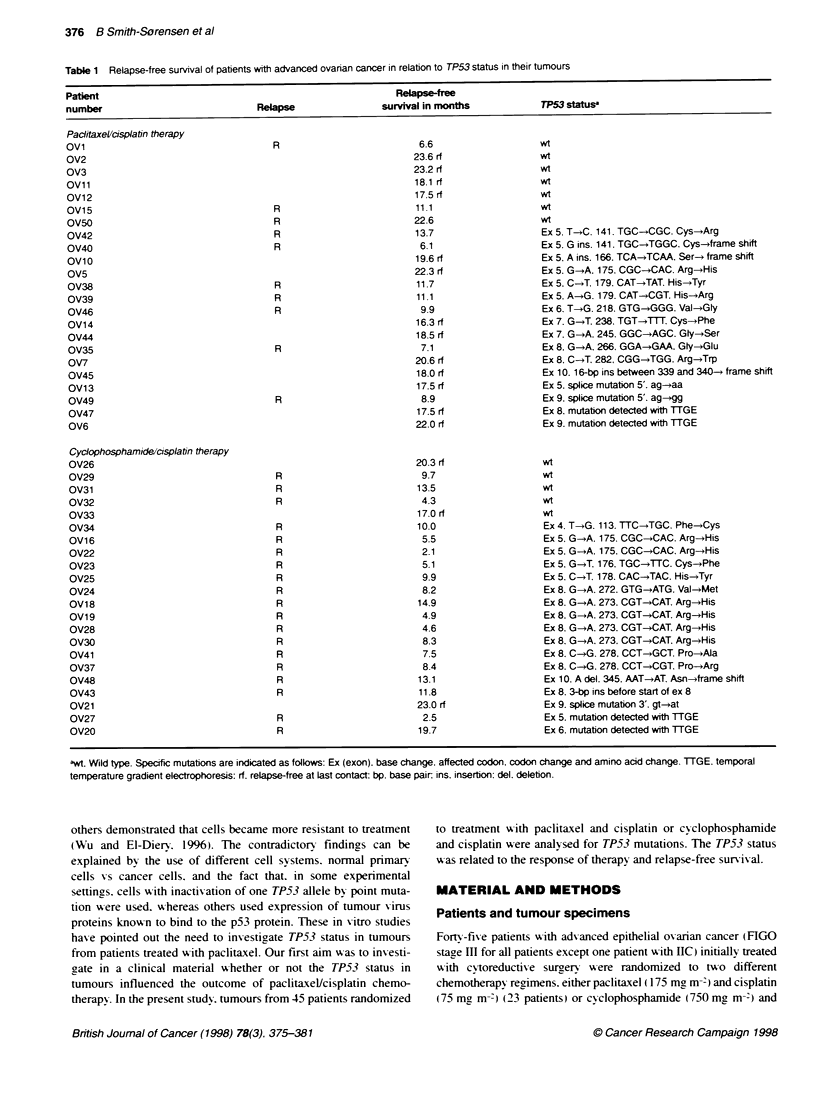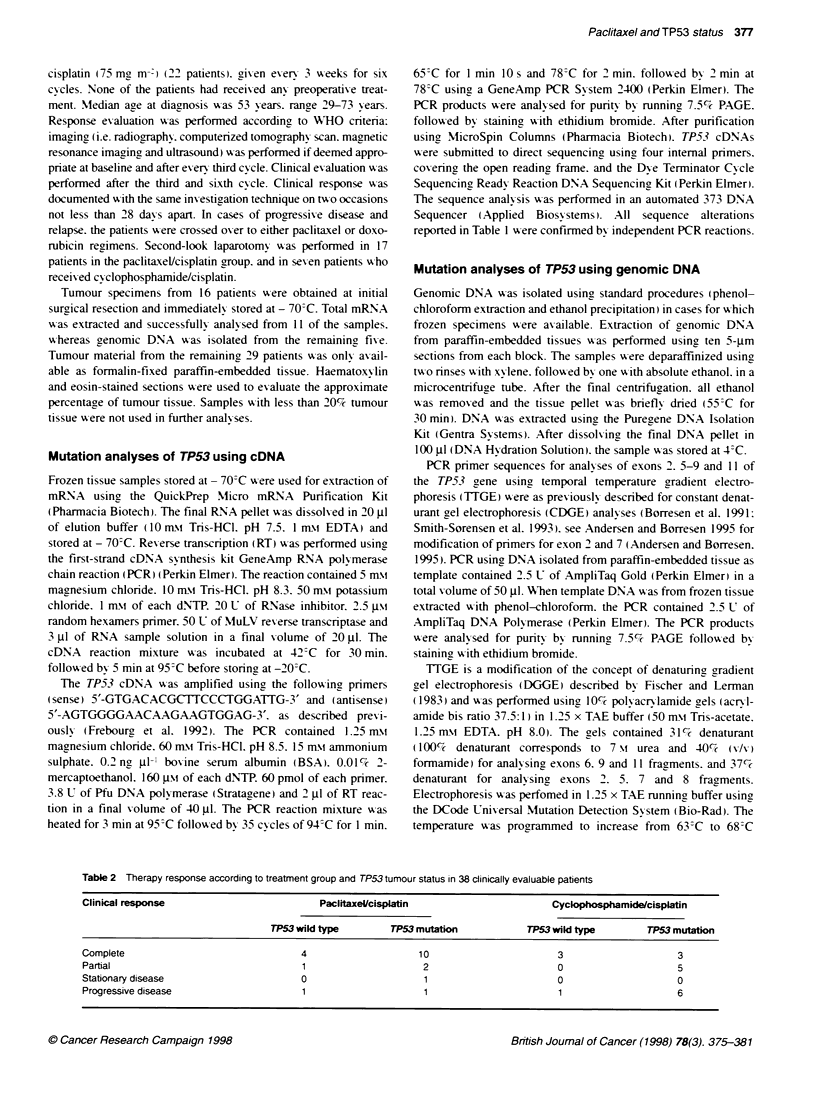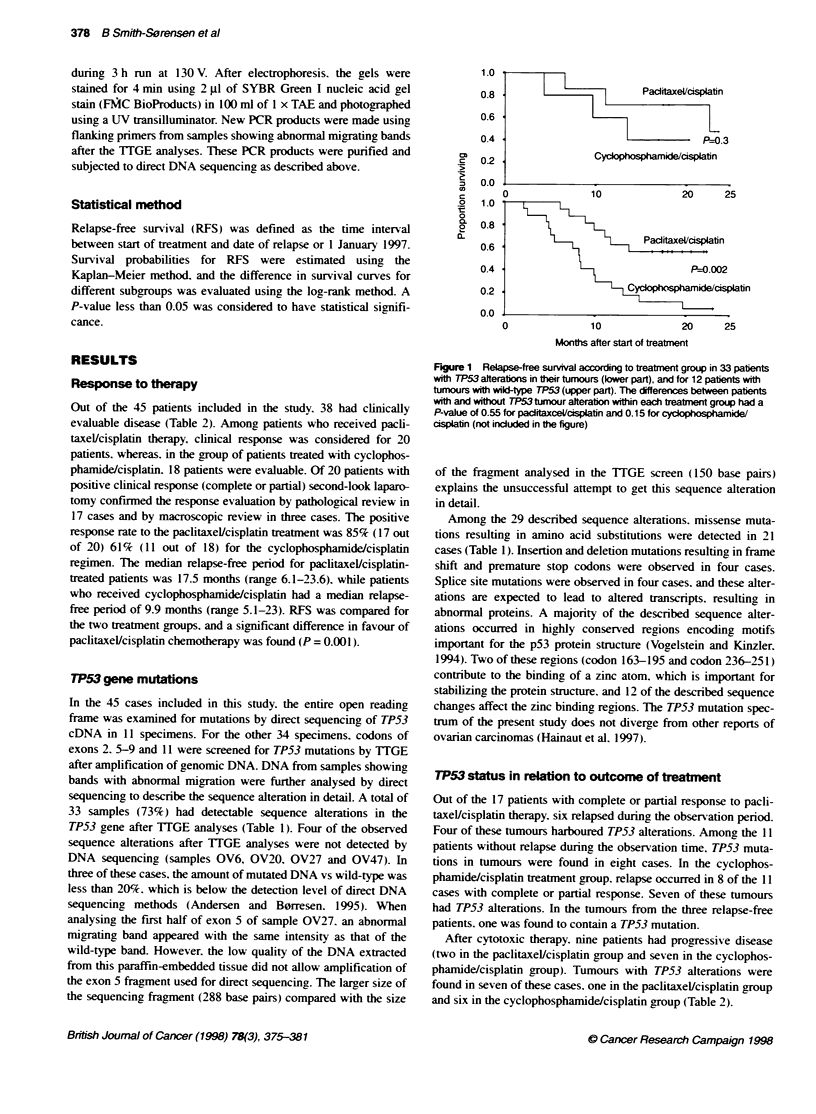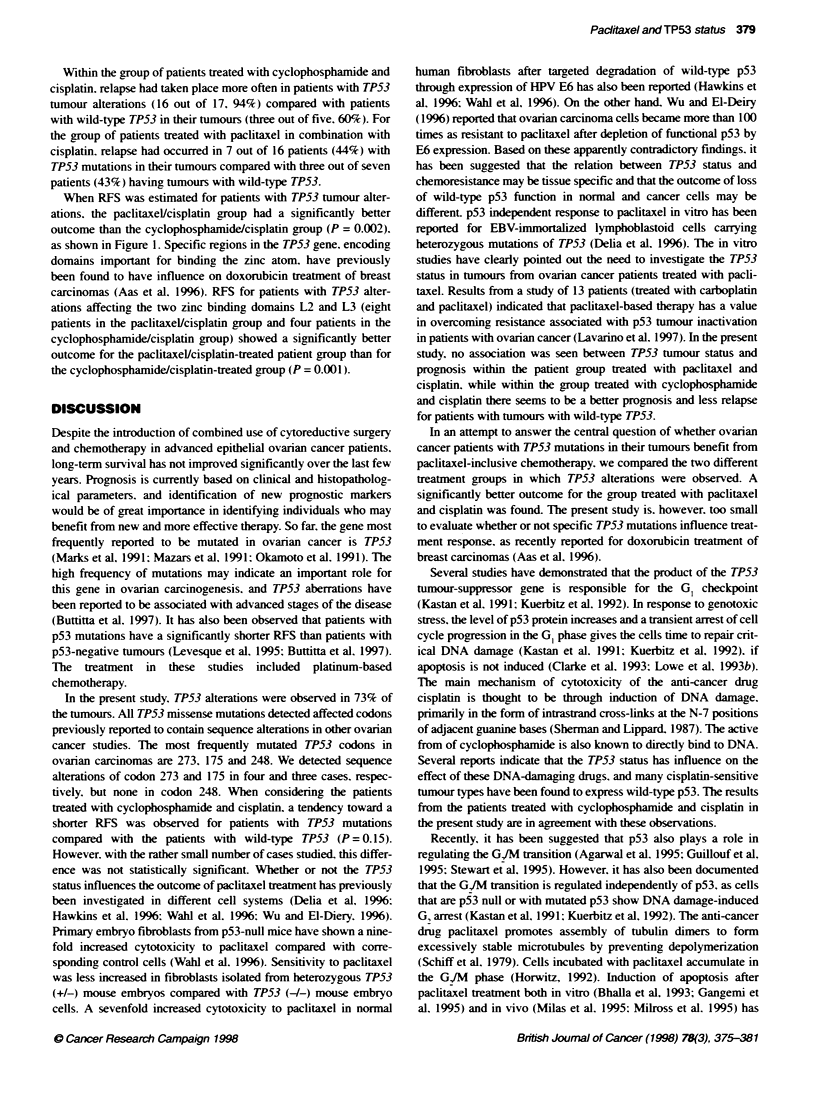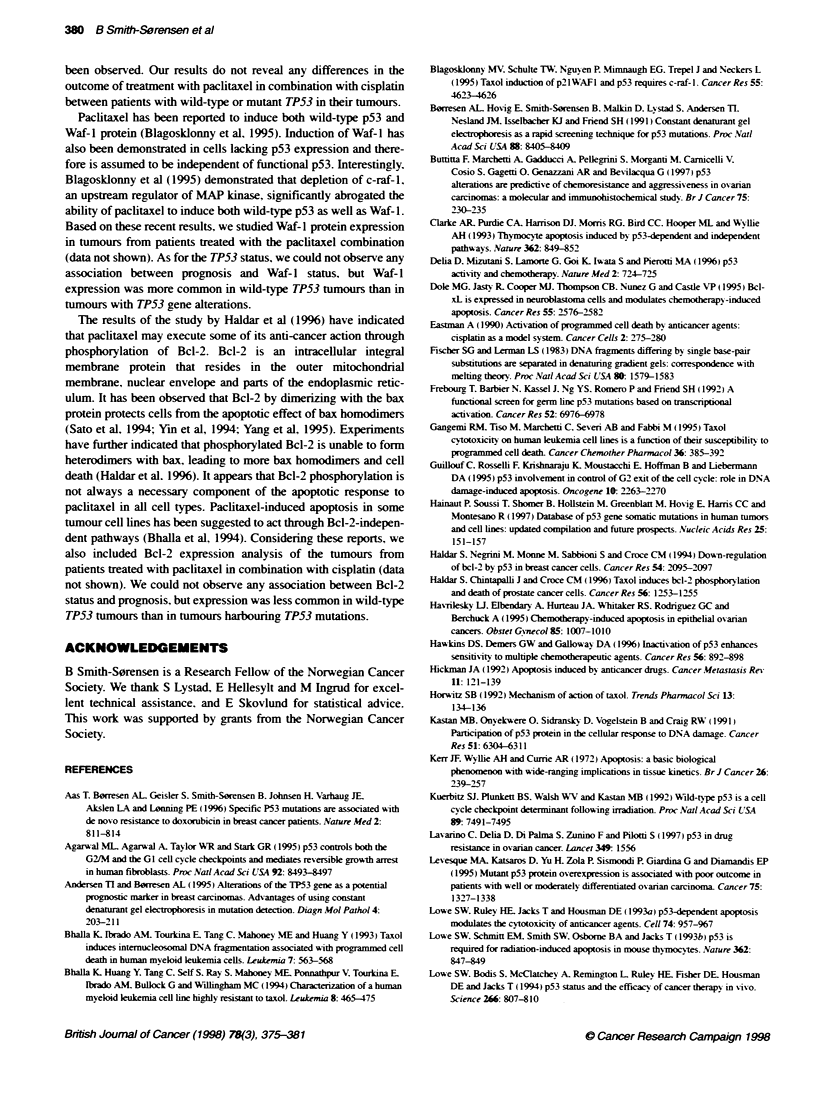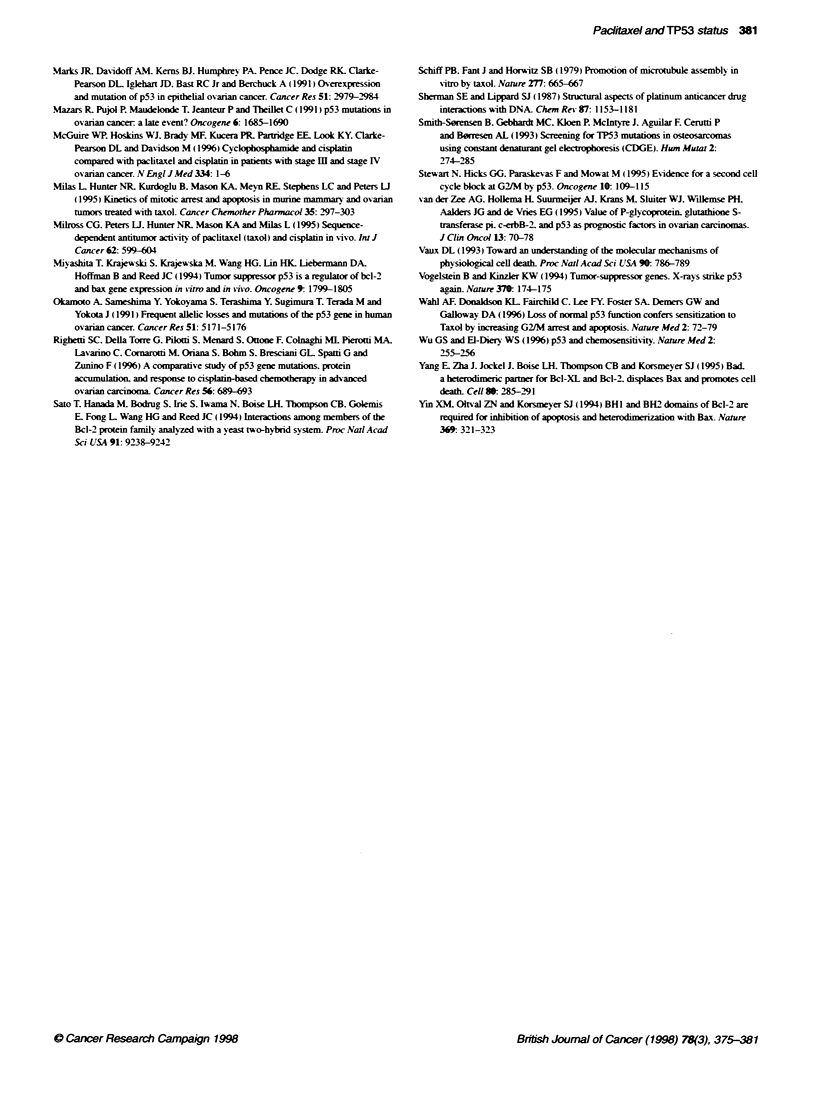# Therapy effect of either paclitaxel or cyclophosphamide combination treatment in patients with epithelial ovarian cancer and relation to TP53 gene status.

**DOI:** 10.1038/bjc.1998.502

**Published:** 1998-08

**Authors:** B. Smith-Sørensen, J. Kaern, R. Holm, A. Dørum, C. Tropé, A. L. Børresen-Dale

**Affiliations:** Department of Genetics, Institute for Cancer Research, The Norwegian Radium Hospital, Oslo.

## Abstract

Cell death after treatment with chemotherapy is exerted by activation of apoptosis, and the p53 protein has been shown to actively participate in this process. This recent focus on TP53 status as a possible determinant of cancer therapy response has raised the question of whether or not mutations in the TP53 gene have an influence on paclitaxel therapy. The TP53 status has been analysed at the DNA level in tumours from 45 ovarian cancer patients randomized to treatment with paclitaxel and cisplatin or cyclophosphamide and cisplatin. Therapy response was obtained for 38 patients with clinically evaluable disease after initial surgery. The positive response rate to the paclitaxel/cisplatin therapy was 85% vs 61% for the patients who received the cyclophosphamide/cisplatin regimen. A significant difference in relapse-free survival in favour of paclitaxel/cisplatin chemotherapy was found (P = 0.001). A total of 33 tumour samples (73%) had detectable sequence alterations in the TP53 gene. When relapse-free survival was estimated for all patients with TP53 alterations in their tumours, a significant better outcome for the paclitaxel/cisplatin group was found compared with the patient group receiving cyclophosphamide and cisplatin therapy (P = 0.002). We did not observe an association between TP53 tumour status and prognosis for patients who received paclitaxel/cisplatin combination treatment, indicating that the effect of this therapy is not influenced by this parameter.


British Journal of Cancer (1998) 78(3). 375–381
© 1998 Cancer Research Campaign

### Therapy effect of either paclitaxel or cyclophosphamide combination treatment in patients with epithelial ovarian cancer and relation to *TP53* gene status

**B Smith-Sørensen[1], J Kærn[2], R Holm[3], A Dørum[4], C Tropé[2] and A-L Børresen-Dale[1]**

[1]Department of Genetics. Institute for Cancer Research. The Norwegian Radium Hospital. 0310 Oslo. Norway: [2]Department of Gynecological Oncology. The Norwegian Radium Hospital. 0310 Oslo. Norway: [3]Department of Pathology. Institute for Cancer Research. The Norwegian Radium Hospital. 0310 Oslo. Norway: [4]Unit of Medical Genetics. Department of Oncology. The Norwegian Radium Hospital. 0310 Oslo. Norway

**Summary** Cell death after treatment with chemotherapy is exerted by activation of apoptosis. and the p53 protein has been shown to actively participate in this process. This recent focus on *TP53* status as a possible determinant of cancer therapy response has raised the question of whether or not mutations in the *TP53* gene have an influence on paclitaxel therapy. The *TP53* status has been analysed at the DNA level in tumours from 45 ovarian cancer patients randomized to treatment with paclitaxel and cisplatin or cyclophosphamide and cisplatin. Therapy response was obtained for 38 patients with clinically evaluable disease after initial surgery. The positive response rate to the paclitaxel/cisplatin therapy was 85% vs 61% for the patients who received the cyclophosphamide/cisplatin regimen. A significant difference in relapse-free survival in favour of paclitaxel/cisplatin chemotherapy was found ($P$ = 0.001). A total of 33 tumour samples (73%) had detectable sequence alterations in the *TP53* gene. When relapse-free survival was estimated for all patients with *TP53* alterations in their tumours. a significant better outcome for the paclitaxel/cisplatin group was found compared with the patient group receiving cyclophosphamide and cisplatin therapy ($P$ = 0.002). We did not observe an association between *TP53* tumour status and prognosis for patients who received paclitaxel/cisplatin combination treatment. indicating that the effect of this therapy is not influenced by this parameter.

**Keywords:** *TP53*: paclitaxel: ovarian cancer

Two-thirds of patients with epithelial ovarian cancer are diagnosed with advanced stages. Treatment includes surgical resection of the primary tumour and metastases to reduce tumour volume. followed by cytotoxic chemotherapy. As many as 60–73% of these ovarian carcinomas are responsive to drug regimens. including the DNA-binding agents cyclophosphamide and cisplatin. or the microtubuli-stabilizing agent paclitaxel together with cisplatin (McGuire et al. 1996). The effectiveness of cancer chemotherapy is restricted by failure of some tumours to respond and by the appearance of resistant cell populations in patients who relapse after initial response. Understanding resistance to chemotherapy is dependent on the elucidation of the molecular mechanisms by which anti-cancer drugs induce cell death (Kerr et al. 1972: Vaux. 1993). It has been shown in several types of carcinomas that cell death after treatment with chemotherapy involves activation of apoptosis (Eastman. 1990: Hickman. 1992). Exposure of the cells to both platinum analogues or taxanes is associated with morphological changes in apoptosis (Bhalla et al. 1993: Havrilesky et al. 1995).

Apoptosis is a genetically controlled process. and a variety of genes have been identified coding for inducers or inhibitors of this process. The tumour-suppressor protein p53 has been shown to exert marked effects on apoptosis in some cells in addition to its involvement in the control of the cell cycle and in DNA repair. Multiple studies have revealed that wild-type p53 protein and members of the Bcl-2 protein family (including Bcl-2 and Bax) undergo complex regulative interactions during the modulation of programmed cell death. Wild-type p53 has been shown to induce an up-regulation of *Bax* mRNA (Miyashita et al. 1994) and a concomitant down-regulation of *Bcl-2* mRNA (Haldar et al. 1994) that correlates to the induction of programmed cell death (Miyashita et al. 1994). It has been reported that cells functionally deficient in p53 or with elevated levels of either Bcl-2 or Bcl-xL are relatively resistant to cytotoxic agents. such as platinum analogues (Lowe et al. 1993*a*. 1994: Dole et al. 1995).

Alterations of the *TP53* gene. either as a result of point mutations or deletions. are the most frequent abnormalities described in human cancers. Abnormal p53 protein has been reported to be associated with shorter disease-free survival and poor clinical outcome of ovarian cancer patients (Levesque et al. 1995: van der Zee et al. 1995). A significant association has been reported between *TP53* missense mutations and tumours resistance to cisplatin-based therapy (Righetti et al. 1996).

During the last years. the inclusion of paclitaxel in the treatment of advanced ovarian cancer has extended the disease-free survival period. The fact that more than 50% of stage III and IV epithelial ovarian cancers are reported to have *TP53* sequence alterations (Hainaut et al. 1997). and the recent focus on *TP53* status as a possible determinant of cancer therapy response. raised the question of whether or not the *TP53* gene has an influence on paclitaxel therapy. Several groups have used various cell systems to investigate the topic (Delia et al. 1996: Hawkins et al. 1996: Wahl et al. 1996: Wu and El-Diery. 1996). Some authors could demonstrate an increased paclitaxel sensitivity for cells harbouring inactivated p53 protein (Hawkins et al. 1996: Wahl et al. 1996). whereas

*Received 27 October 1997*
*Revised 13 January 1998*
*Accepted 21 January 1998*

*Correspondence to:* A-L Børresen-Dale

Table 1 Relapse-free survival of patients with advanced ovarian cancer in relation to *TP53* status in their tumours

| Patient number | Relapse | Relapse-free survival in months | *TP53* status[a] |
|---|---|---|---|
| *Paclitaxel/cisplatin therapy* | | | |
| OV1 | R | 6.6 | wt |
| OV2 | | 23.6 rf | wt |
| OV3 | | 23.2 rf | wt |
| OV11 | | 18.1 rf | wt |
| OV12 | | 17.5 rf | wt |
| OV15 | R | 11.1 | wt |
| OV50 | R | 22.6 | wt |
| OV42 | R | 13.7 | Ex 5. T→C. 141. TGC→CGC. Cys→Arg |
| OV40 | R | 6.1 | Ex 5. G ins. 141. TGC→TGGC. Cys→frame shift |
| OV10 | | 19.6 rf | Ex 5. A ins. 166. TCA→TCAA. Ser→ frame shift |
| OV5 | | 22.3 rf | Ex 5. G→A. 175. CGC→CAC. Arg→His |
| OV38 | R | 11.7 | Ex 5. C→T. 179. CAT→TAT. His→Tyr |
| OV39 | R | 11.1 | Ex 5. A→G. 179. CAT→CGT. His→Arg |
| OV46 | R | 9.9 | Ex 6. T→G. 218. GTG→GGG. Val→Gly |
| OV14 | | 16.3 rf | Ex 7. G→T. 238. TGT→TTT. Cys→Phe |
| OV44 | | 18.5 rf | Ex 7. G→A. 245. GGC→AGC. Gly→Ser |
| OV35 | R | 7.1 | Ex 8. G→A. 266. GGA→GAA. Gly→Glu |
| OV7 | | 20.6 rf | Ex 8. C→T. 282. CGG→TGG. Arg→Trp |
| OV45 | | 18.0 rf | Ex 10. 16-bp ins between 339 and 340→ frame shift |
| OV13 | | 17.5 rf | Ex 5. splice mutation 5′. ag→aa |
| OV49 | R | 8.9 | Ex 9. splice mutation 5′. ag→gg |
| OV47 | | 17.5 rf | Ex 8. mutation detected with TTGE |
| OV6 | | 22.0 rf | Ex 9. mutation detected with TTGE |
| *Cyclophosphamide/cisplatin therapy* | | | |
| OV26 | | 20.3 rf | wt |
| OV29 | R | 9.7 | wt |
| OV31 | R | 13.5 | wt |
| OV32 | R | 4.3 | wt |
| OV33 | | 17.0 rf | wt |
| OV34 | R | 10.0 | Ex 4. T→G. 113. TTC→TGC. Phe→Cys |
| OV16 | R | 5.5 | Ex 5. G→A. 175. CGC→CAC. Arg→His |
| OV22 | R | 2.1 | Ex 5. G→A. 175. CGC→CAC. Arg→His |
| OV23 | R | 5.1 | Ex 5. G→T. 176. TGC→TTC. Cys→Phe |
| OV25 | R | 9.9 | Ex 5. C→T. 178. CAC→TAC. His→Tyr |
| OV24 | R | 8.2 | Ex 8. G→A. 272. GTG→ATG. Val→Met |
| OV18 | R | 14.9 | Ex 8. G→A. 273. CGT→CAT. Arg→His |
| OV19 | R | 4.9 | Ex 8. G→A. 273. CGT→CAT. Arg→His |
| OV28 | R | 4.6 | Ex 8. G→A. 273. CGT→CAT. Arg→His |
| OV30 | R | 8.3 | Ex 8. G→A. 273. CGT→CAT. Arg→His |
| OV41 | R | 7.5 | Ex 8. C→G. 278. CCT→GCT. Pro→Ala |
| OV37 | R | 8.4 | Ex 8. C→G. 278. CCT→CGT. Pro→Arg |
| OV48 | R | 13.1 | Ex 10. A del. 345. AAT→AT. Asn→frame shift |
| OV43 | R | 11.8 | Ex 8. 3-bp ins before start of ex 8 |
| OV21 | | 23.0 rf | Ex 9. splice mutation 3′. gt→at |
| OV27 | R | 2.5 | Ex 5. mutation detected with TTGE |
| OV20 | R | 19.7 | Ex 6. mutation detected with TTGE |

[a]wt. Wild type. Specific mutations are indicated as follows: Ex (exon). base change. affected codon. codon change and amino acid change. TTGE. temporal temperature gradient electrophoresis: rf. relapse-free at last contact: bp. base pair: ins. insertion: del. deletion.

others demonstrated that cells became more resistant to treatment (Wu and El-Diery. 1996). The contradictory findings can be explained by the use of different cell systems. normal primary cells vs cancer cells. and the fact that. in some experimental settings. cells with inactivation of one *TP53* allele by point mutation were used. whereas others used expression of tumour virus proteins known to bind to the p53 protein. These in vitro studies have pointed out the need to investigate *TP53* status in tumours from patients treated with paclitaxel. Our first aim was to investigate in a clinical material whether or not the *TP53* status in tumours influenced the outcome of paclitaxel/cisplatin chemotherapy. In the present study. tumours from 45 patients randomized to treatment with paclitaxel and cisplatin or cyclophosphamide and cisplatin were analysed for *TP53* mutations. The *TP53* status was related to the response of therapy and relapse-free survival.

### MATERIAL AND METHODS

### Patients and tumour specimens

Forty-five patients with advanced epithelial ovarian cancer (FIGO stage III for all patients except one patient with IIC) initially treated with cytoreductive surgery were randomized to two different chemotherapy regimens. either paclitaxel (175 mg m$^{-2}$) and cisplatin (75 mg m$^{-2}$) (23 patients) or cyclophosphamide (750 mg m$^{-2}$) and

© Cancer Research Campaign 1998

cisplatin (75 mg m⁻²) (22 patients), given every 3 weeks for six cycles. None of the patients had received any preoperative treatment. Median age at diagnosis was 53 years, range 29–73 years. Response evaluation was performed according to WHO criteria; imaging (i.e. radiography, computerized tomography scan, magnetic resonance imaging and ultrasound) was performed if deemed appropriate at baseline and after every third cycle. Clinical evaluation was performed after the third and sixth cycle. Clinical response was documented with the same investigation technique on two occasions not less than 28 days apart. In cases of progressive disease and relapse, the patients were crossed over to either paclitaxel or doxorubicin regimens. Second-look laparotomy was performed in 17 patients in the paclitaxel/cisplatin group, and in seven patients who received cyclophosphamide/cisplatin.

Tumour specimens from 16 patients were obtained at initial surgical resection and immediately stored at – 70°C. Total mRNA was extracted and successfully analysed from 11 of the samples, whereas genomic DNA was isolated from the remaining five. Tumour material from the remaining 29 patients was only available as formalin-fixed paraffin-embedded tissue. Haematoxylin and eosin-stained sections were used to evaluate the approximate percentage of tumour tissue. Samples with less than 20% tumour tissue were not used in further analyses.

### Mutation analyses of *TP53* using cDNA

Frozen tissue samples stored at – 70°C were used for extraction of mRNA using the QuickPrep Micro mRNA Purification Kit (Pharmacia Biotech). The final RNA pellet was dissolved in 20 µl of elution buffer (10 mM Tris-HCl, pH 7.5, 1 mM EDTA) and stored at – 70°C. Reverse transcription (RT) was performed using the first-strand cDNA synthesis kit GeneAmp RNA polymerase chain reaction (PCR) (Perkin Elmer). The reaction contained 5 mM magnesium chloride, 10 mM Tris-HCl, pH 8.3, 50 mM potassium chloride, 1 mM of each dNTP, 20 U of RNase inhibitor, 2.5 µM random hexamers primer, 50 U of MuLV reverse transcriptase and 3 µl of RNA sample solution in a final volume of 20 µl. The cDNA reaction mixture was incubated at 42°C for 30 min, followed by 5 min at 95°C before storing at –20°C.

The *TP53* cDNA was amplified using the following primers (sense) 5′-GTGACACGCTTCCCTGGATTG-3′ and (antisense) 5′-AGTGGGGAACAAGAAGTGGAG-3′, as described previously (Frebourg et al, 1992). The PCR contained 1.25 mM magnesium chloride, 60 mM Tris-HCl, pH 8.5, 15 mM ammonium sulphate, 0.2 ng µl⁻¹ bovine serum albumin (BSA), 0.01% 2-mercaptoethanol, 160 µM of each dNTP, 60 pmol of each primer, 3.8 U of Pfu DNA polymerase (Stratagene) and 2 µl of RT reaction in a final volume of 40 µl. The PCR reaction mixture was heated for 3 min at 95°C followed by 35 cycles of 94°C for 1 min, 65°C for 1 min 10 s and 78°C for 2 min, followed by 2 min at 78°C using a GeneAmp PCR System 2400 (Perkin Elmer). The PCR products were analysed for purity by running 7.5% PAGE, followed by staining with ethidium bromide. After purification using MicroSpin Columns (Pharmacia Biotech), *TP53* cDNAs were submitted to direct sequencing using four internal primers, covering the open reading frame, and the Dye Terminator Cycle Sequencing Ready Reaction DNA Sequencing Kit (Perkin Elmer). The sequence analysis was performed in an automated 373 DNA Sequencer (Applied Biosystems). All sequence alterations reported in Table 1 were confirmed by independent PCR reactions.

### Mutation analyses of *TP53* using genomic DNA

Genomic DNA was isolated using standard procedures (phenol–chloroform extraction and ethanol precipitation) in cases for which frozen specimens were available. Extraction of genomic DNA from paraffin-embedded tissues was performed using ten 5-µm sections from each block. The samples were deparaffinized using two rinses with xylene, followed by one with absolute ethanol, in a microcentrifuge tube. After the final centrifugation, all ethanol was removed and the tissue pellet was briefly dried (55°C for 30 min). DNA was extracted using the Puregene DNA Isolation Kit (Gentra Systems). After dissolving the final DNA pellet in 100 µl (DNA Hydration Solution), the sample was stored at 4°C.

PCR primer sequences for analyses of exons 2, 5–9 and 11 of the *TP53* gene using temporal temperature gradient electrophoresis (TTGE) were as previously described for constant denaturant gel electrophoresis (CDGE) analyses (Børresen et al, 1991; Smith-Sørensen et al, 1993), see Andersen and Børresen 1995 for modification of primers for exon 2 and 7 (Andersen and Børresen, 1995). PCR using DNA isolated from paraffin-embedded tissue as template contained 2.5 U of AmpliTaq Gold (Perkin Elmer) in a total volume of 50 µl. When template DNA was from frozen tissue extracted with phenol–chloroform, the PCR contained 2.5 U of AmpliTaq DNA Polymerase (Perkin Elmer). The PCR products were analysed for purity by running 7.5% PAGE followed by staining with ethidium bromide.

TTGE is a modification of the concept of denaturing gradient gel electrophoresis (DGGE) described by Fischer and Lerman (1983) and was performed using 10% polyacrylamide gels (acrylamide bis ratio 37.5:1) in 1.25 × TAE buffer (50 mM Tris-acetate, 1.25 mM EDTA, pH 8.0). The gels contained 31% denaturant (100% denaturant corresponds to 7 M urea and 40% (v/v) formamide) for analysing exons 6, 9 and 11 fragments, and 37% denaturant for analysing exons 2, 5, 7 and 8 fragments. Electrophoresis was perfomed in 1.25 × TAE running buffer using the DCode Universal Mutation Detection System (Bio-Rad). The temperature was programmed to increase from 63°C to 68°C

**Table 2** Therapy response according to treatment group and *TP53* tumour status in 38 clinically evaluable patients

| Clinical response | Paclitaxel/cisplatin | | Cyclophosphamide/cisplatin | |
|---|---|---|---|---|
| | *TP53* wild type | *TP53* mutation | *TP53* wild type | *TP53* mutation |
| Complete | 4 | 10 | 3 | 3 |
| Partial | 1 | 2 | 0 | 5 |
| Stationary disease | 0 | 1 | 0 | 0 |
| Progressive disease | 1 | 1 | 1 | 6 |

during 3 h run at 130 V. After electrophoresis, the gels were stained for 4 min using 2 µl of SYBR Green I nucleic acid gel stain (FMC BioProducts) in 100 ml of 1 × TAE and photographed using a UV transilluminator. New PCR products were made using flanking primers from samples showing abnormal migrating bands after the TTGE analyses. These PCR products were purified and subjected to direct DNA sequencing as described above.

### Statistical method

Relapse-free survival (RFS) was defined as the time interval between start of treatment and date of relapse or 1 January 1997. Survival probabilities for RFS were estimated using the Kaplan–Meier method, and the difference in survival curves for different subgroups was evaluated using the log-rank method. A $P$-value less than 0.05 was considered to have statistical significance.

### RESULTS

### Response to therapy

Out of the 45 patients included in the study, 38 had clinically evaluable disease (Table 2). Among patients who received paclitaxel/cisplatin therapy, clinical response was considered for 20 patients, whereas, in the group of patients treated with cyclophosphamide/cisplatin, 18 patients were evaluable. Of 20 patients with positive clinical response (complete or partial) second-look laparotomy confirmed the response evaluation by pathological review in 17 cases and by macroscopic review in three cases. The positive response rate to the paclitaxel/cisplatin treatment was 85% (17 out of 20) 61% (11 out of 18) for the cyclophosphamide/cisplatin regimen. The median relapse-free period for paclitaxel/cisplatin-treated patients was 17.5 months (range 6.1–23.6), while patients who received cyclophosphamide/cisplatin had a median relapse-free period of 9.9 months (range 5.1–23). RFS was compared for the two treatment groups, and a significant difference in favour of paclitaxel/cisplatin chemotherapy was found ($P = 0.001$).

### *TP53* gene mutations

In the 45 cases included in this study, the entire open reading frame was examined for mutations by direct sequencing of *TP53* cDNA in 11 specimens. For the other 34 specimens, codons of exons 2, 5–9 and 11 were screened for *TP53* mutations by TTGE after amplification of genomic DNA. DNA from samples showing bands with abnormal migration were further analysed by direct sequencing to describe the sequence alteration in detail. A total of 33 samples (73%) had detectable sequence alterations in the *TP53* gene after TTGE analyses (Table 1). Four of the observed sequence alterations after TTGE analyses were not detected by DNA sequencing (samples OV6, OV20, OV27 and OV47). In three of these cases, the amount of mutated DNA vs wild-type was less than 20%, which is below the detection level of direct DNA sequencing methods (Andersen and Børresen, 1995). When analysing the first half of exon 5 of sample OV27, an abnormal migrating band appeared with the same intensity as that of the wild-type band. However, the low quality of the DNA extracted from this paraffin-embedded tissue did not allow amplification of the exon 5 fragment used for direct sequencing. The larger size of the sequencing fragment (288 base pairs) compared with the size of the fragment analysed in the TTGE screen (150 base pairs) explains the unsuccessful attempt to get this sequence alteration in detail.

**Figure 1** Relapse-free survival according to treatment group in 33 patients with *TP53* alterations in their tumours (lower part), and for 12 patients with tumours with wild-type *TP53* (upper part). The differences between patients with and without *TP53* tumour alteration within each treatment group had a $P$-value of 0.55 for paclitaxcel/cisplatin and 0.15 for cyclophosphamide/cisplatin (not included in the figure)

Among the 29 described sequence alterations, missense mutations resulting in amino acid substitutions were detected in 21 cases (Table 1). Insertion and deletion mutations resulting in frame shift and premature stop codons were observed in four cases. Splice site mutations were observed in four cases, and these alterations are expected to lead to altered transcripts, resulting in abnormal proteins. A majority of the described sequence alterations occurred in highly conserved regions encoding motifs important for the p53 protein structure (Vogelstein and Kinzler, 1994). Two of these regions (codon 163–195 and codon 236–251) contribute to the binding of a zinc atom, which is important for stabilizing the protein structure, and 12 of the described sequence changes affect the zinc binding regions. The *TP53* mutation spectrum of the present study does not diverge from other reports of ovarian carcinomas (Hainaut et al, 1997).

### *TP53* status in relation to outcome of treatment

Out of the 17 patients with complete or partial response to paclitaxel/cisplatin therapy, six relapsed during the observation period. Four of these tumours harboured *TP53* alterations. Among the 11 patients without relapse during the observation time, *TP53* mutations in tumours were found in eight cases. In the cyclophosphamide/cisplatin treatment group, relapse occurred in 8 of the 11 cases with complete or partial response. Seven of these tumours had *TP53* alterations. In the tumours from the three relapse-free patients, one was found to contain a *TP53* mutation.

After cytotoxic therapy, nine patients had progressive disease (two in the paclitaxel/cisplatin group and seven in the cyclophosphamide/cisplatin group). Tumours with *TP53* alterations were found in seven of these cases, one in the paclitaxel/cisplatin group and six in the cyclophosphamide/cisplatin group (Table 2).

© Cancer Research Campaign 1998

Within the group of patients treated with cyclophosphamide and cisplatin, relapse had taken place more often in patients with *TP53* tumour alterations (16 out of 17, 94%) compared with patients with wild-type *TP53* in their tumours (three out of five, 60%). For the group of patients treated with paclitaxel in combination with cisplatin, relapse had occurred in 7 out of 16 patients (44%) with *TP53* mutations in their tumours compared with three out of seven patients (43%) having tumours with wild-type *TP53*.

When RFS was estimated for patients with *TP53* tumour alterations, the paclitaxel/cisplatin group had a significantly better outcome than the cyclophosphamide/cisplatin group ($P = 0.002$), as shown in Figure 1. Specific regions in the *TP53* gene, encoding domains important for binding the zinc atom, have previously been found to have influence on doxorubicin treatment of breast carcinomas (Aas et al, 1996). RFS for patients with *TP53* alterations affecting the two zinc binding domains L2 and L3 (eight patients in the paclitaxel/cisplatin group and four patients in the cyclophosphamide/cisplatin group) showed a significantly better outcome for the paclitaxel/cisplatin-treated patient group than for the cyclophosphamide/cisplatin-treated group ($P = 0.001$).

### DISCUSSION

Despite the introduction of combined use of cytoreductive surgery and chemotherapy in advanced epithelial ovarian cancer patients, long-term survival has not improved significantly over the last few years. Prognosis is currently based on clinical and histopathological parameters, and identification of new prognostic markers would be of great importance in identifying individuals who may benefit from new and more effective therapy. So far, the gene most frequently reported to be mutated in ovarian cancer is *TP53* (Marks et al, 1991; Mazars et al, 1991; Okamoto et al, 1991). The high frequency of mutations may indicate an important role for this gene in ovarian carcinogenesis, and *TP53* aberrations have been reported to be associated with advanced stages of the disease (Buttitta et al, 1997). It has also been observed that patients with p53 mutations have a significantly shorter RFS than patients with p53-negative tumours (Levesque et al, 1995; Buttitta et al, 1997). The treatment in these studies included platinum-based chemotherapy.

In the present study, *TP53* alterations were observed in 73% of the tumours. All *TP53* missense mutations detected affected codons previously reported to contain sequence alterations in other ovarian cancer studies. The most frequently mutated *TP53* codons in ovarian carcinomas are 273, 175 and 248. We detected sequence alterations of codon 273 and 175 in four and three cases, respectively, but none in codon 248. When considering the patients treated with cyclophosphamide and cisplatin, a tendency toward a shorter RFS was observed for patients with *TP53* mutations compared with the patients with wild-type *TP53* ($P = 0.15$). However, with the rather small number of cases studied, this difference was not statistically significant. Whether or not the *TP53* status influences the outcome of paclitaxel treatment has previously been investigated in different cell systems (Delia et al, 1996; Hawkins et al, 1996; Wahl et al, 1996; Wu and El-Diery, 1996). Primary embryo fibroblasts from p53-null mice have shown a ninefold increased cytotoxicity to paclitaxel compared with corresponding control cells (Wahl et al, 1996). Sensitivity to paclitaxel was less increased in fibroblasts isolated from heterozygous *TP53* (+/–) mouse embryos compared with *TP53* (–/–) mouse embryo cells. A sevenfold increased cytotoxicity to paclitaxel in normal human fibroblasts after targeted degradation of wild-type p53 through expression of HPV E6 has also been reported (Hawkins et al, 1996; Wahl et al, 1996). On the other hand, Wu and El-Deiry (1996) reported that ovarian carcinoma cells became more than 100 times as resistant to paclitaxel after depletion of functional p53 by E6 expression. Based on these apparently contradictory findings, it has been suggested that the relation between *TP53* status and chemoresistance may be tissue specific and that the outcome of loss of wild-type p53 function in normal and cancer cells may be different. p53 independent response to paclitaxel in vitro has been reported for EBV-immortalized lymphoblastoid cells carrying heterozygous mutations of *TP53* (Delia et al, 1996). The in vitro studies have clearly pointed out the need to investigate the *TP53* status in tumours from ovarian cancer patients treated with paclitaxel. Results from a study of 13 patients (treated with carboplatin and paclitaxel) indicated that paclitaxel-based therapy has a value in overcoming resistance associated with p53 tumour inactivation in patients with ovarian cancer (Lavarino et al, 1997). In the present study, no association was seen between *TP53* tumour status and prognosis within the patient group treated with paclitaxel and cisplatin, while within the group treated with cyclophosphamide and cisplatin there seems to be a better prognosis and less relapse for patients with tumours with wild-type *TP53*.

In an attempt to answer the central question of whether ovarian cancer patients with *TP53* mutations in their tumours benefit from paclitaxel-inclusive chemotherapy, we compared the two different treatment groups in which *TP53* alterations were observed. A significantly better outcome for the group treated with paclitaxel and cisplatin was found. The present study is, however, too small to evaluate whether or not specific *TP53* mutations influence treatment response, as recently reported for doxorubicin treatment of breast carcinomas (Aas et al, 1996).

Several studies have demonstrated that the product of the *TP53* tumour-suppressor gene is responsible for the $G_1$ checkpoint (Kastan et al, 1991; Kuerbitz et al, 1992). In response to genotoxic stress, the level of p53 protein increases and a transient arrest of cell cycle progression in the $G_1$ phase gives the cells time to repair critical DNA damage (Kastan et al, 1991; Kuerbitz et al, 1992), if apoptosis is not induced (Clarke et al, 1993; Lowe et al, 1993*b*). The main mechanism of cytotoxicity of the anti-cancer drug cisplatin is thought to be through induction of DNA damage, primarily in the form of intrastrand cross-links at the N-7 positions of adjacent guanine bases (Sherman and Lippard, 1987). The active from of cyclophosphamide is also known to directly bind to DNA. Several reports indicate that the *TP53* status has influence on the effect of these DNA-damaging drugs, and many cisplatin-sensitive tumour types have been found to express wild-type p53. The results from the patients treated with cyclophosphamide and cisplatin in the present study are in agreement with these observations.

Recently, it has been suggested that p53 also plays a role in regulating the $G_2/M$ transition (Agarwal et al, 1995; Guillouf et al, 1995; Stewart et al, 1995). However, it has also been documented that the $G_2/M$ transition is regulated independently of p53, as cells that are p53 null or with mutated p53 show DNA damage-induced $G_2$ arrest (Kastan et al, 1991; Kuerbitz et al, 1992). The anti-cancer drug paclitaxel promotes assembly of tubulin dimers to form excessively stable microtubules by preventing depolymerization (Schiff et al, 1979). Cells incubated with paclitaxel accumulate in the $G_2/M$ phase (Horwitz, 1992). Induction of apoptosis after paclitaxel treatment both in vitro (Bhalla et al, 1993; Gangemi et al, 1995) and in vivo (Milas et al, 1995; Milross et al, 1995) has

© Cancer Research Campaign 1998

been observed. Our results do not reveal any differences in the outcome of treatment with paclitaxel in combination with cisplatin between patients with wild-type or mutant *TP53* in their tumours.

Paclitaxel has been reported to induce both wild-type p53 and Waf-1 protein (Blagosklonny et al, 1995). Induction of Waf-1 has also been demonstrated in cells lacking p53 expression and therefore is assumed to be independent of functional p53. Interestingly, Blagosklonny et al (1995) demonstrated that depletion of c-raf-1, an upstream regulator of MAP kinase, significantly abrogated the ability of paclitaxel to induce both wild-type p53 as well as Waf-1. Based on these recent results, we studied Waf-1 protein expression in tumours from patients treated with the paclitaxel combination (data not shown). As for the *TP53* status, we could not observe any association between prognosis and Waf-1 status, but Waf-1 expression was more common in wild-type *TP53* tumours than in tumours with *TP53* gene alterations.

The results of the study by Haldar et al (1996) have indicated that paclitaxel may execute some of its anti-cancer action through phosphorylation of Bcl-2. Bcl-2 is an intracellular integral membrane protein that resides in the outer mitochondrial membrane, nuclear envelope and parts of the endoplasmic reticulum. It has been observed that Bcl-2 by dimerizing with the bax protein protects cells from the apoptotic effect of bax homodimers (Sato et al, 1994; Yin et al, 1994; Yang et al, 1995). Experiments have further indicated that phosphorylated Bcl-2 is unable to form heterodimers with bax, leading to more bax homodimers and cell death (Haldar et al, 1996). It appears that Bcl-2 phosphorylation is not always a necessary component of the apoptotic response to paclitaxel in all cell types. Paclitaxel-induced apoptosis in some tumour cell lines has been suggested to act through Bcl-2-independent pathways (Bhalla et al, 1994). Considering these reports, we also included Bcl-2 expression analysis of the tumours from patients treated with paclitaxel in combination with cisplatin (data not shown). We could not observe any association between Bcl-2 status and prognosis, but expression was less common in wild-type *TP53* tumours than in tumours harbouring *TP53* mutations.

### ACKNOWLEDGEMENTS

B Smith-Sørensen is a Research Fellow of the Norwegian Cancer Society. We thank S Lystad, E Hellesylt and M Ingrud for excellent technical assistance, and E Skovlund for statistical advice. This work was supported by grants from the Norwegian Cancer Society.

### REFERENCES

Aas T, Børresen AL, Geisler S, Smith-Sørensen B, Johnsen H, Varhaug JE, Akslen LA and Lønning PE (1996) Specific P53 mutations are associated with de novo resistance to doxorubicin in breast cancer patients. *Nature Med* **2**: 811–814

Agarwal ML, Agarwal A, Taylor WR and Stark GR (1995) p53 controls both the G2/M and the G1 cell cycle checkpoints and mediates reversible growth arrest in human fibroblasts. *Proc Natl Acad Sci USA* **92**: 8493–8497

Andersen TI and Børresen AL (1995) Alterations of the TP53 gene as a potential prognostic marker in breast carcinomas. Advantages of using constant denaturant gel electrophoresis in mutation detection. *Diagn Mol Pathol* **4**: 203–211

Bhalla K, Ibrado AM, Tourkina E, Tang C, Mahoney ME and Huang Y (1993) Taxol induces internucleosomal DNA fragmentation associated with programmed cell death in human myeloid leukemia cells. *Leukemia* **7**: 563–568

Bhalla K, Huang Y, Tang C, Self S, Ray S, Mahoney ME, Ponnathpur V, Tourkina E, Ibrado AM, Bullock G and Willingham MC (1994) Characterization of a human myeloid leukemia cell line highly resistant to taxol. *Leukemia* **8**: 465–475

Blagosklonny MV, Schulte TW, Nguyen P, Mimnaugh EG, Trepel J and Neckers L (1995) Taxol induction of p21WAF1 and p53 requires c-raf-1. *Cancer Res* **55**: 4623–4626

Børresen AL, Hovig E, Smith-Sørensen B, Malkin D, Lystad S, Andersen TI, Nesland JM, Isselbacher KJ and Friend SH (1991) Constant denaturant gel electrophoresis as a rapid screening technique for p53 mutations. *Proc Natl Acad Sci USA* **88**: 8405–8409

Buttitta F, Marchetti A, Gadducci A, Pellegrini S, Morganti M, Carnicelli V, Cosio S, Gagetti O, Genazzani AR and Bevilacqua G (1997) p53 alterations are predictive of chemoresistance and aggressiveness in ovarian carcinomas: a molecular and immunohistochemical study. *Br J Cancer* **75**: 230–235

Clarke AR, Purdie CA, Harrison DJ, Morris RG, Bird CC, Hooper ML and Wyllie AH (1993) Thymocyte apoptosis induced by p53-dependent and independent pathways. *Nature* **362**: 849–852

Delia D, Mizutani S, Lamorte G, Goi K, Iwata S and Pierotti MA (1996) p53 activity and chemotherapy. *Nature Med* **2**: 724–725

Dole MG, Jasty R, Cooper MJ, Thompson CB, Nunez G and Castle VP (1995) Bcl-xL is expressed in neuroblastoma cells and modulates chemotherapy-induced apoptosis. *Cancer Res* **55**: 2576–2582

Eastman A (1990) Activation of programmed cell death by anticancer agents: cisplatin as a model system. *Cancer Cells* **2**: 275–280

Fischer SG and Lerman LS (1983) DNA fragments differing by single base-pair substitutions are separated in denaturing gradient gels: correspondence with melting theory. *Proc Natl Acad Sci USA* **80**: 1579–1583

Frebourg T, Barbier N, Kassel J, Ng YS, Romero P and Friend SH (1992) A functional screen for germ line p53 mutations based on transcriptional activation. *Cancer Res* **52**: 6976–6978

Gangemi RM, Tiso M, Marchetti C, Severi AB and Fabbi M (1995) Taxol cytotoxicity on human leukemia cell lines is a function of their susceptibility to programmed cell death. *Cancer Chemother Pharmacol* **36**: 385–392

Guillouf C, Rosselli F, Krishnaraju K, Moustacchi E, Hoffman B and Liebermann DA (1995) p53 involvement in control of G2 exit of the cell cycle: role in DNA damage-induced apoptosis. *Oncogene* **10**: 2263–2270

Hainaut P, Soussi T, Shomer B, Hollstein M, Greenblatt M, Hovig E, Harris CC and Montesano R (1997) Database of p53 gene somatic mutations in human tumors and cell lines: updated compilation and future prospects. *Nucleic Acids Res* **25**: 151–157

Haldar S, Negrini M, Monne M, Sabbioni S and Croce CM (1994) Down-regulation of bcl-2 by p53 in breast cancer cells. *Cancer Res* **54**: 2095–2097

Haldar S, Chintapalli J and Croce CM (1996) Taxol induces bcl-2 phosphorylation and death of prostate cancer cells. *Cancer Res* **56**: 1253–1255

Havrilesky LJ, Elbendary A, Hurteau JA, Whitaker RS, Rodriguez GC and Berchuck A (1995) Chemotherapy-induced apoptosis in epithelial ovarian cancers. *Obstet Gynecol* **85**: 1007–1010

Hawkins DS, Demers GW and Galloway DA (1996) Inactivation of p53 enhances sensitivity to multiple chemotherapeutic agents. *Cancer Res* **56**: 892–898

Hickman JA (1992) Apoptosis induced by anticancer drugs. *Cancer Metastasis Rev* **11**: 121–139

Horwitz SB (1992) Mechanism of action of taxol. *Trends Pharmacol Sci* **13**: 134–136

Kastan MB, Onyekwere O, Sidransky D, Vogelstein B and Craig RW (1991) Participation of p53 protein in the cellular response to DNA damage. *Cancer Res* **51**: 6304–6311

Kerr JF, Wyllie AH and Currie AR (1972) Apoptosis: a basic biological phenomenon with wide-ranging implications in tissue kinetics. *Br J Cancer* **26**: 239–257

Kuerbitz SJ, Plunkett BS, Walsh WV and Kastan MB (1992) Wild-type p53 is a cell cycle checkpoint determinant following irradiation. *Proc Natl Acad Sci USA* **89**: 7491–7495

Lavarino C, Delia D, Di Palma S, Zunino F and Pilotti S (1997) p53 in drug resistance in ovarian cancer. *Lancet* **349**: 1556

Levesque MA, Katsaros D, Yu H, Zola P, Sismondi P, Giardina G and Diamandis EP (1995) Mutant p53 protein overexpression is associated with poor outcome in patients with well or moderately differentiated ovarian carcinoma. *Cancer* **75**: 1327–1338

Lowe SW, Ruley HE, Jacks T and Housman DE (1993*a*) p53-dependent apoptosis modulates the cytotoxicity of anticancer agents. *Cell* **74**: 957–967

Lowe SW, Schmitt EM, Smith SW, Osborne BA and Jacks T (1993*b*) p53 is required for radiation-induced apoptosis in mouse thymocytes. *Nature* **362**: 847–849

Lowe SW, Bodis S, McClatchey A, Remington L, Ruley HE, Fisher DE, Housman DE and Jacks T (1994) p53 status and the efficacy of cancer therapy in vivo. *Science* **266**: 807–810

© Cancer Research Campaign 1998

Marks JR, Davidoff AM, Kerns BJ, Humphrey PA, Pence JC, Dodge RK, Clarke-Pearson DL, Iglehart JD, Bast RC Jr and Berchuck A (1991) Overexpression and mutation of p53 in epithelial ovarian cancer. *Cancer Res* **51**: 2979–2984

Mazars R, Pujol P, Maudelonde T, Jeanteur P and Theillet C (1991) p53 mutations in ovarian cancer: a late event? *Oncogene* **6**: 1685–1690

McGuire WP, Hoskins WJ, Brady MF, Kucera PR, Partridge EE, Look KY, Clarke-Pearson DL and Davidson M (1996) Cyclophosphamide and cisplatin compared with paclitaxel and cisplatin in patients with stage III and stage IV ovarian cancer. *N Engl J Med* **334**: 1–6

Milas L, Hunter NR, Kurdoglu B, Mason KA, Meyn RE, Stephens LC and Peters LJ (1995) Kinetics of mitotic arrest and apoptosis in murine mammary and ovarian tumors treated with taxol. *Cancer Chemother Pharmacol* **35**: 297–303

Milross CG, Peters LJ, Hunter NR, Mason KA and Milas L (1995) Sequence-dependent antitumor activity of paclitaxel (taxol) and cisplatin in vivo. *Int J Cancer* **62**: 599–604

Miyashita T, Krajewski S, Krajewska M, Wang HG, Lin HK, Liebermann DA, Hoffman B and Reed JC (1994) Tumor suppressor p53 is a regulator of bcl-2 and bax gene expression *in vitro* and *in vivo*. *Oncogene* **9**: 1799–1805

Okamoto A, Sameshima Y, Yokoyama S, Terashima Y, Sugimura T, Terada M and Yokota J (1991) Frequent allelic losses and mutations of the p53 gene in human ovarian cancer. *Cancer Res* **51**: 5171–5176

Righetti SC, Della Torre G, Pilotti S, Menard S, Ottone F, Colnaghi MI, Pierotti MA, Lavarino C, Cornarotti M, Oriana S, Bohm S, Bresciani GL, Spatti G and Zunino F (1996) A comparative study of p53 gene mutations, protein accumulation, and response to cisplatin-based chemotherapy in advanced ovarian carcinoma. *Cancer Res* **56**: 689–693

Sato T, Hanada M, Bodrug S, Irie S, Iwama N, Boise LH, Thompson CB, Golemis E, Fong L, Wang HG and Reed JC (1994) Interactions among members of the Bcl-2 protein family analyzed with a yeast two-hybrid system. *Proc Natl Acad Sci USA* **91**: 9238–9242

Schiff PB, Fant J and Horwitz SB (1979) Promotion of microtubule assembly in vitro by taxol. *Nature* **277**: 665–667

Sherman SE and Lippard SJ (1987) Structural aspects of platinum anticancer drug interactions with DNA. *Chem Rev* **87**: 1153–1181

Smith-Sørensen B, Gebhardt MC, Kloen P, McIntyre J, Aguilar F, Cerutti P and Børresen AL (1993) Screening for TP53 mutations in osteosarcomas using constant denaturant gel electrophoresis (CDGE). *Hum Mutat* **2**: 274–285

Stewart N, Hicks GG, Paraskevas F and Mowat M (1995) Evidence for a second cell cycle block at G2/M by p53. *Oncogene* **10**: 109–115

van der Zee AG, Hollema H, Suurmeijer AJ, Krans M, Sluiter WJ, Willemse PH, Aalders JG and de Vries EG (1995) Value of P-glycoprotein, glutathione S-transferase pi, c-erbB-2, and p53 as prognostic factors in ovarian carcinomas. *J Clin Oncol* **13**: 70–78

Vaux DL (1993) Toward an understanding of the molecular mechanisms of physiological cell death. *Proc Natl Acad Sci USA* **90**: 786–789

Vogelstein B and Kinzler KW (1994) Tumor-suppressor genes. X-rays strike p53 again. *Nature* **370**: 174–175

Wahl AF, Donaldson KL, Fairchild C, Lee FY, Foster SA, Demers GW and Galloway DA (1996) Loss of normal p53 function confers sensitization to Taxol by increasing G2/M arrest and apoptosis. *Nature Med* **2**: 72–79

Wu GS and El-Diery WS (1996) p53 and chemosensitivity. *Nature Med* **2**: 255–256

Yang E, Zha J, Jockel J, Boise LH, Thompson CB and Korsmeyer SJ (1995) Bad, a heterodimeric partner for Bcl-XL and Bcl-2, displaces Bax and promotes cell death. *Cell* **80**: 285–291

Yin XM, Oltval ZN and Korsmeyer SJ (1994) BH1 and BH2 domains of Bcl-2 are required for inhibition of apoptosis and heterodimerization with Bax. *Nature* **369**: 321–323

© Cancer Research Campaign 1998